# Baseline Statin Use Appears to Protect Against Severe COVID-19

**DOI:** 10.1093/ofid/ofae697

**Published:** 2024-11-26

**Authors:** Eric A Meyerowitz, Arthur Y Kim

**Affiliations:** Division of Infectious Diseases, Department of Medicine, Montefiore Medical Center, Bronx, New York, USA; Albert Einstein College of Medicine, Bronx, New York, USA; Division of Infectious Diseases, Department of Medicine, Massachusetts General Hospital, Boston, Massachusetts, USA; Harvard Medical School, Boston, Massachusetts, USA

The potential role for statins in the treatment and prevention of severe coronavirus infections was proposed long before the coronavirus disease 2019 (COVID-19) pandemic [1]. The rationale for statin use was based on nonrandomized data suggesting possible benefit for other noncoronavirus respiratory viral infections like influenza [2–4] and, also, because of a theoretical benefit in dampening specific coronavirus-triggered inflammatory pathways [5, 6]. Several nonrandomized studies have suggested a possible association of baseline statin use with decreased risk of severe COVID-19 outcomes, but this result may be confounded by factors related to their use [7–9]. Zanni et al. provide the highest quality evidence to date suggesting a possible benefit of baseline statin use in reducing severe COVID-19 outcomes [10].

Zanni et al. cleverly leveraged the REPRIEVE trial database to examine the relationship between baseline statin use and COVID-19 outcomes [10]. REPRIEVE, arguably the most important nonantiretroviral randomized trial for people with HIV in the modern era, found a lower risk of cardiovascular outcomes for people with HIV with low to moderate cardiovascular disease risk who received pitavastatin vs placebo [11].

The REPRIEVE investigators measured the effect on COVID-19 severity in 6905 trial participants remaining in follow-up as of January 2020, 95% of whom had been on treatment (or placebo) for more than a year at the start of analysis [10]. By the end of analysis, 84% and 83% in the pitavastatin and placebo groups, respectively, had received COVID-19 vaccines, with a median follow-up of 3.3 years. The 1701 COVID-19 diagnoses included 1684 symptomatic cases and 15 deaths (8 in the pitavastatin group and 7 in the placebo group). The risk of serious COVID-19 (defined as resulting in hospitalization or death) was 5.75% and 8.05% in the pitavastatin and placebo groups, respectively (relative risk, 0.73; 95% CI, 0.52–1.03), representing a reduction of 27% in the pitavastatin group. This effect fell within the hypothesized range, though significance was not met because there were too few severe events due to the impact of vaccines (the prespecified target was 200 serious events, with just 117 by the end of the trial). By the end of the study, 83% of the entire cohort had received COVID-19 vaccines, greatly reducing the risk of serious COVID-19 events thereafter (hazard ratio for serious COVID-19 before vs after vaccination, 0.27; 95% CI, 0.14–0.53; *P* < .0001). Of the 117 total serious COVID-19 events, 100 (85.47%) occurred before vaccination.

Together with the body of observational data, these results suggest that baseline statin use reduced the likelihood of severe COVID-19 outcomes, especially before the role of widespread vaccination. Importantly, the largest randomized controlled trial examining the role of statin addition at the time of COVID-19 diagnosis in critically ill patients found no benefit [12]. The REMAP-CAP group used a randomized open-label design to study simvastatin 80 mg daily vs no statin for critically ill patients with COVID-19 who were not on statins at baseline. The study included 2739 critically ill patients who were receiving high-level supplemental oxygen support (high-flow nasal canula, noninvasive or invasive mechanical ventilation) and/or vasopressor support at enrollment. While the study found a higher number of organ support–free days in the simvastatin group compared with the control group, the finding did not meet the prespecified threshold for superiority. Of note, a subgroup analysis found that fewer patients not receiving mechanical ventilation at baseline who received simvastatin progressed to mechanical ventilation, extracorporeal membrane oxygenation (ECMO), or death compared with those not receiving simvastatin (37.0% vs 42.5%). Taken together with the results from the REPRIEVE secondary analysis, the accumulating data suggest that baseline statin use—but not addition of statins after the onset of critical disease—may be effective in preventing severe COVID-19 outcomes.

Timing of administration of treatments for COVID-19 has been critical, with antivirals having a role early and anti-inflammatories typically being deployed later in the disease course [13]. Statins, if they are indeed acting through an anti-inflammatory pathway, may dampen the inflammation before maximal inflammation has been unleashed. These results did not indicate any effect on the development of incident COVID-19, which is not surprising given that a protective effect of statins on acquisition of a respiratory viral infection would have less biologic plausibility.

In the desperation of the early COVID-19 pandemic days, many unproven therapies were administered to patients in an attempt to save lives [14]. The herculean efforts of the medical community and the openness of patients to enrolling in randomized trials have led to critical breakthroughs in understanding the science behind COVID-19 pathophysiology and elucidating effective treatments. This study team should be commended for harnessing the infrastructure of a large ongoing randomized trial to answer a key question posed in the early days of the pandemic. While the exact role of statins is still being studied, the REPRIEVE results strongly suggest a benefit to baseline statin use in preventing severe disease. The possibility that this benefit might apply to other noncoronavirus respiratory viral infections with severe outcomes needs to be studied. The adaptation of clinical trial platforms such as REPRIEVE, REMAP-CAP, and others provided critical beacons of information amid a fog of unknowns; this is one lesson learned from this pandemic that will help us prepare for the next one.
